# Window-Based Channel Impulse Response Prediction for Time-Varying Ultra-Wideband Channels

**DOI:** 10.1371/journal.pone.0164944

**Published:** 2016-12-19

**Authors:** A. M. Al-Samman, M. H. Azmi, T. A. Rahman, I. Khan, M. N. Hindia, A. Fattouh

**Affiliations:** 1 Department Wireless Communication Centre, Faculty of Electrical Engineering, Universiti Teknologi Malaysia, 81310, Johor, Malaysia; 2 Department of Electrical Engineering, Islamic University Madinah, Madinah, Saudi Arabia; 3 Department of Computer Sciences, Faculty of Computing and Information Technology, King Abdulaziz University (KAU), Jeddah, Saudi Arabia; University of Kent, UNITED KINGDOM

## Abstract

This work proposes channel impulse response (CIR) prediction for time-varying ultra-wideband (UWB) channels by exploiting the fast movement of channel taps within delay bins. Considering the sparsity of UWB channels, we introduce a window-based CIR (WB-CIR) to approximate the high temporal resolutions of UWB channels. A recursive least square (RLS) algorithm is adopted to predict the time evolution of the WB-CIR. For predicting the future WB-CIR tap of window *w*_*k*_, three RLS filter coefficients are computed from the observed WB-CIRs of the left *w*_*k*−1_, the current *w*_*k*_ and the right *w*_*k*+1_ windows. The filter coefficient with the lowest RLS error is used to predict the future WB-CIR tap. To evaluate our proposed prediction method, UWB CIRs are collected through measurement campaigns in outdoor environments considering line-of-sight (LOS) and non-line-of-sight (NLOS) scenarios. Under similar computational complexity, our proposed method provides an improvement in prediction errors of approximately 80% for LOS and 63% for NLOS scenarios compared with a conventional method.

## Introduction

Channel predictions are applied in wireless communication systems with fast time-varying channels because the channel state information (CSI) obtained through channel estimations becomes outdated shortly after its acquisition [1–3]. The prediction of wireless channels constitutes an important building block of advanced wireless mobile transceivers [4,5]. In adaptive orthogonal frequency division multiplexing (OFDM) systems, for instance, the predicted CSI is used to determine the guard time interval of OFDM sub-channels for the upcoming transmission frame [6]. In [7], a prediction method for flat fading channels using only the important scattered characteristics was developed. Prediction techniques for OFDM systems were investigated in [8]. Whereas OFDM is an efficient modulation scheme for broadband communications [9,10], the OFDM sub-channels are assumed to be flat fading [11]. In [12], a channel predictor was proposed that consists of an envelope predictor to trace the slow envelope variation and a phase slope detector to follow the phase changes for each multipath cluster. Studies on predicting wireless channels with large system bandwidths were considered in [13–15]. A comparative study of time domain prediction techniques for wideband systems was performed in [13]. For ultra-wideband (UWB) channels, the work of [14] reported that UWB channels are predictable, and their proposed prediction algorithm has been shown to achieve more than 70% of the matched filter output energies for line-of-sight (LOS) and 40% for non-line-of-sight (NLOS) scenarios.

In this work, a prediction method for UWB channels is developed by considering the fast movement of channel impulse response (CIR) taps across delay bins and the sparsity of UWB channels. This prediction method is used to predict the CIR tap in the time domain based on measurements in an outdoor environment. The prediction method applies non-overlapping timing windows to group the CIR delay bins and break the rapid channel variation across delays, hence reducing the complication of the prediction. Each window is then represented using only one channel tap called a window tap. The strongest channel tap among all grouped taps in the window is selected as the window tap. We refer to this CIR as window-based CIR (WB-CIR). Considering the channel tap correlation across window delay bins, the prediction of the future WB-CIR tap in window *k* (i.e., *w*_*k*_) begins with the computation of three recursive least square (RLS) filter coefficients. The three coefficients are computed independently using the observed WB-CIR channel taps from the left *w*_*k*−1_, the current *w*_*k*_ and the right *w*_*k*+1_ windows. The filter coefficient with the lowest RLS error and its corresponding observed window taps are used to predict the future WB-CIR tap of window *k*. Note that our prediction method is general in the sense that other adaptive filters can also be applied. To evaluate our proposed method, the prediction errors between the predicted and measured WB-CIRs from field test measurements are presented.

The rest of this paper is organized as follows; the channel model is described in Section 2. The proposed prediction method is presented in Section 3. The measurement setups are described in Section 4. Section 5 provides the evaluation criterion. Section 6 gives details of a complexity evaluation. The results and analysis are discussed in Section 7. The limitation and future work are given in Section 8. Finally, we conclude the paper in Section 9.

## Channel Model

The impulse response of UWB channels is represented using a tap delay line model [14,16]:

$$h\left(\tau \right)=\sum_{i=1}^{L}a_{i}\delta \left(\tau -m_{i}\right),$$ (1)

where *a*_*i*_ and *m*_*i*_ are the *i-th* path gain and delay, respectively. For time-varying channels, (1) can be modified as [14]:

$$h\left(t,\tau \right)=\sum_{i=1}^{L}a_{i}\left(t\right)\delta \left(\tau -m_{i}\left(t\right)\right),$$ (2)

where *t* is either the time or the spatial location. Assuming that the transmitter moves at a constant velocity away from the receiver, we can convert between the time and spatial location. The time and spatial location are used interchangeably in this work. For real communication systems, the space and delay must be sampled. By applying the transformation *t* = *p*Δ*d*, (2) in discrete form can be expressed as:

$$h\left(p\Delta d,lT_{s}\right)=\sum_{i=1}^{L}a_{i}\left(p\Delta d\right)\delta \left(lT_{s}-m_{i}\left(p\Delta d\right)\right),$$ (3)

where *l* is the delay index from 1,2, …, L, and *T*_*S*_ is the sampling period over the delay. In addition, *p* ∈ [1,2,…,*P*] is the position or location index for the transmitter or receiver antenna movement, and Δ*d* is the sampling distance of the antennas. Using shorthand *l* = *lT*_*s*_ and *p* = *p*Δ*d* for simplification, the discrete UWB channel model in (3) becomes:

$$h\left(p,l\right)=\sum_{i=1}^{L}a_{i}\left(p\right)\delta \left(l-m_{i}\left(p\right)\right)$$ (4)

### Proposed Prediction Method

We consider the problem of modeling the evolution of UWB channels in (4). Specifically, the aim is to model and predict the CIR at the next Tx–Rx location index *p* + 1, given *M* observed CIRs at location indexes *p*, *p* − 1,…, *p* − *M* + 1.

Our proposed prediction method is described as follows: We first group the delay bins using *K* non-overlapping windows. For UWB channels with high temporal resolutions, some of the resolvable delay bins are empty; hence, the delay bins containing multipath components (MPCs) are interspersed with empty delay bins, which leads to a sparse CIR [17,18]. Considering the sparsity of the UWB channel, we simplify the CIR of (4) by approximating the grouped taps in each window using only one tap (called the window tap) with the delay parameter of the last bin in the window (called the window delay). Among all channel taps grouped in each window, the strongest tap gain is selected as the window tap. As a result, the UWB channel in (4) can be simplified as:

$$h\left(p,l\right)\approx h_{w}\left(p,l\right)=\sum_{i=1}^{K}a_{w_{i}}\left(p\right)\delta \left(l-m_{w_{i}}\left(p\right)\right),$$ (5)

where $a_{w_{i}}$ and $m_{w_{i}}$ are the *w*_*i*_-th window tap gain and delay, respectively. We refer to (5) as the window-based channel impulse response. The way to calculate the *K* parameter is discussed as follows. If the total number of bins in a particular CIR is *L* and the size of each window is *s = 1/B*, *K* represents the number of windows in CIR and is calculated by *K = 1+*⌈*(L*-1)/*s*⌉, where ⌈*x*⌉ denotes the smallest integer larger than or equal to *x*; the 1 is added to account for the first window that represents the direct path and dominant path for LOS and NLOS, respectively. When producing the WB-CIR, the size of the first window is always set to one bin to contain the direct path in LOS scenarios or the most dominant path in NLOS scenarios. Regarding the remaining windows, an equal window size is used. This window arrangement makes the channel tap in the first window independent of the channel taps in the other windows across all spatial locations. This is because the CIR in (4) is normalized with respect to the direct path in LOS scenarios or to the most dominant path in NLOS scenarios. Hence, the first window will be represented using unity tap gain (i.e., $a_{w_{1}}=1$) when the WB-CIRs of (5) are generated from the measured CIRs for all location indices. As a result, no prediction is required for $a_{w_{1}}$. Using the WB-CIR in (5), the evolution of UWB channels is modeled as an autoregressive (AR) process of order *M*.

The relative movement of the transmitter (Tx) and receiver (Rx) over several wavelengths introduces variation in the delays of MPCs, thus switching an MPC from one delay bin to another [19]. As a result, when predicting the future WB-CIR at *p* + 1 of window *w*_*k*_, which corresponds to the strongest MPC, there is a likelihood that the future WB-CIR tap is the MPC coming from the left *w*_*k*−1_ or right *w*_*k*+1_ window. Hence, the AR process, modeling the evolution of UWB channels, should consider this possibility. The channel tap at location index *p* + 1 for window *w*_*k*_ can be predicted using:

$${\tilde{a}}_{w_{k}}\left(p+1\right)=C_{A}^{H}\left(p\right).a_{A}\left(p\right),$$ (6)

for *k* = 2,3,…, *K*, and *A* ∈ {*w*_*k*−1_,*w*_*k*_,*w*_*k*+1_} denotes the left, current and right window, respectively. The vector *a*_*A*_(*p*) = [*a*_*A*_(*p*),*a*_*A*_(*p* − 1),…,*a*_*A*_(*p* − *M* + 1)]^*T*^ corresponds to the observed *M* window taps of the present *p* and *M-1* previous location indices from window *A*, whereas the vector **C**_*A*_(*p*) = [*c*_*A*_(0),*c*_*A*_(1),……,*c*_*A*_(*M* − 1)]^*T*^ represents the prediction coefficient. The superscripts *H* and *T* denote the Hermitian and transpose operators, respectively. Here, we use ${\tilde{a}}_{w_{i}}$ and $a_{w_{i}}$ to distinguish between the tap gain of window *i* obtained from the prediction and measurement, respectively.

Considering the channel tap correlation across delay bins, the key in the proposed prediction in (6) is the usage of three filter coefficients, where for *k* = 2, only two filter coefficients are computed for the current window *w*_2_ and the right window *w*_3_. This is because the left window *w*_1_ contains only the unchanged and unity channel gain of the direct path for LOS or the dominant path for NLOS in tracking the evolution of the channel tap in window *w*_*k*_. The three coefficients are computed independently and simultaneously using *M* observed window taps each from windows *w*_*k*−1_,*w*_*k*_ and *w*_*k*+1_. In this work, the recursive least square algorithm is used to compute the prediction filter coefficients **C**_*A*_(*p*) with initial values:

$$C_{A}^{}\left(p\right)=\left[0,0,\ldots .,0\right]$$ (7)

The resulting update equation for **C**_*A*_(*p*) is given by:

$$C_{A}^{}\left(p\right)=C_{A}^{}\left(p-1\right)+K_{A}^{H}\left(p-1\right).e\left(p\right),$$ (8)

where *e(p)* is the prediction error defined as:

$$e\left(p\right)=a_{A}\left(p\right)-C_{A}^{H}\left(p-1\right)a_{A}\left(p-1\right),$$ (9)

and the gain factor *K*_*A*_(*p*) of RLS is defined as [8]:

$$K_{A}^{}\left(p\right)=\frac{{\text{G}}_{A}\left(P-1\right)a_{A}^{}\left(P\right)}{\gamma +a_{A}^{H}\left(P\right)\times {\text{G}}_{A}\left(P-1\right)\times a_{A}\left(P\right)}$$ (10)

The **G**_*A*_(*P*−1) in (10) is the inverse of the *M* × *M* sample covariance with an initial value of *ζ*^−1^*I*, where *ζ* is a small positive constant (we chose *ζ* = 0.1), and *I* is the identity matrix. It is calculated recursively using [13]:

$${\text{G}}_{A}\left(P\right)=\frac{\left[1-K_{A}^{}\left(p\right)a_{A}^{H}\left(P\right)\right]{\text{G}}_{A}\left(P-1\right)}{\gamma },$$ (11)

where *γ* ∈ {0,1} is the forgetting factor that controls the influence of observed CIRs in the prediction. In this work, *γ* = 0.7 is used because it provides good prediction. Finally, once all three filter coefficients for *A* ∈ {*w*_*k*−1_,*w*_*k*_,*w*_*k*+1_} are obtained when executing the RLS algorithms under *S* iterations, the final updated coefficient that produces the lowest prediction error of (9) and its corresponding observed window taps are used for predicting the future WB-CIR at location index *p+1* in (6). The experimental CIR data are collected from the time domain UWB channel measurements to evaluate the proposed prediction method, which is described in the next section.

### Measurement Setup and Environment

**Fig 1:**
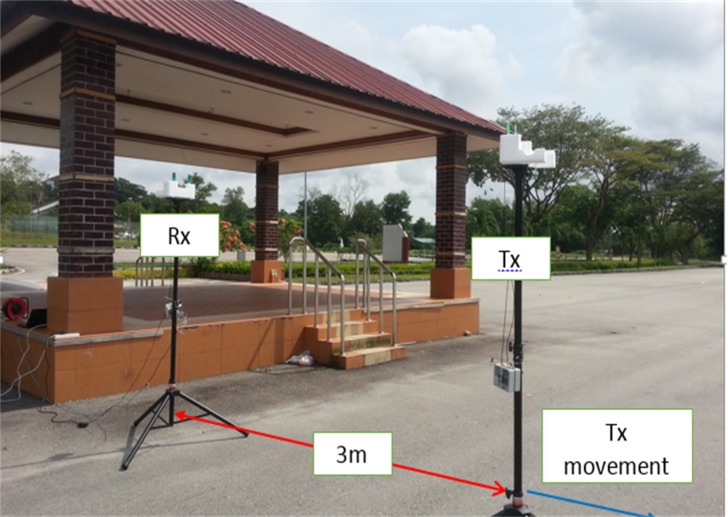
The Channel Measurement Setup for the Open Environment.

**Fig 2:**
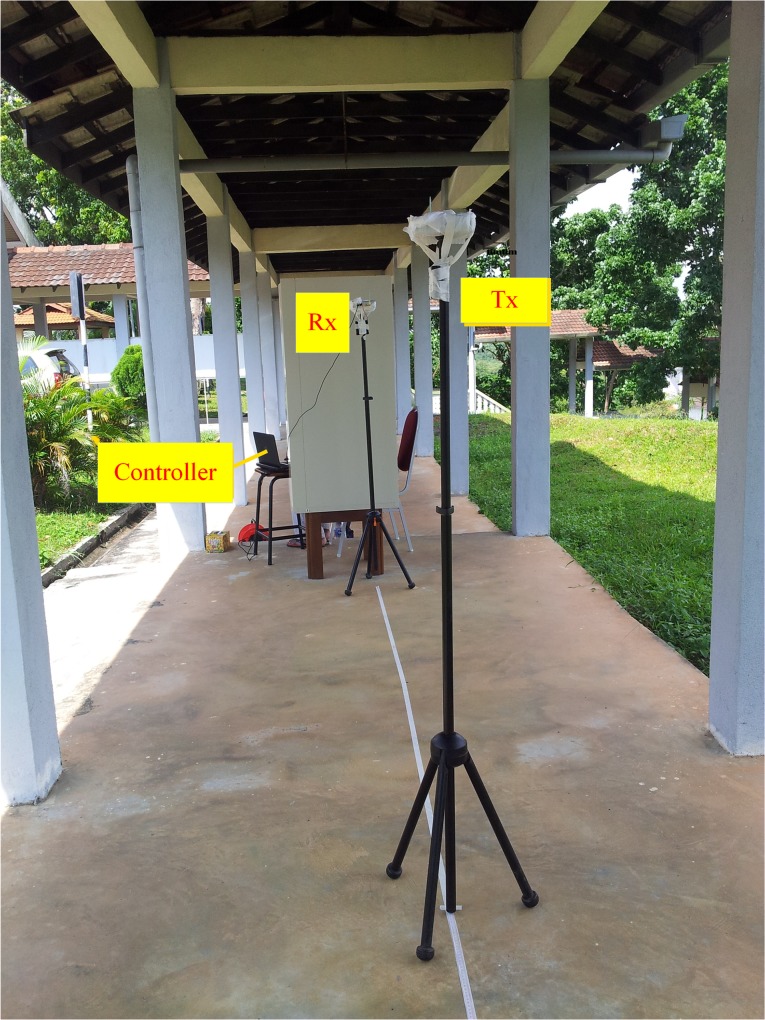
The Channel Measurement Setup for the Outdoor Corridor Environment.

The UWB CIRs are collected from time domain measurements conducted in outdoor environments for LOS and NLOS scenarios by using a pair of PulsON^®^410 transceivers equipped with vertically polarized omnidirectional wideband (3.1–10.6 GHz) dipole antennas (Figs A and B in S1 Text). The details of the measurement devices can be found in [20]. The parameters for the measurement setup are summarized in Table 1. The measurements were taken at two different sites inside Universiti Teknologi Malaysia. The first measurement run was carried out in an open place where the receiver was placed in front of the viewing platform, whereas the transmitter was moved away from the receiver as illustrated in Fig 1. At every transmitter movement of 1 cm, the CIRs were recorded starting from the Tx–Rx separation distance of 3.0 m up to 3.31 m. The operator of the setup sat behind a brick column to ensure a stationary environment. In the NLOS scenario, the directed path (LOS path) was blocked by a human obstacle with a height of 1.6 m standing between the receiver and transmitter antennas at a distance of 2 m from the receiver. The second set of measurements was taken in an outdoor corridor that represents a rich multipath environment, as shown in Fig 2. This outdoor environment consists of a concrete floor and roofing, wooden columns used to support the roofing, and many cars in the parking area. A grounded metallic sheet was placed between the person operating the attached computer system and the setup itself to ensure a stationary environment. The receiver antenna was located at a fixed point adjacent to the metallic sheet, whereas the transmitter part was allowed to move. The transmission antenna height was 2.5 m, and the height of the receiver antenna was 1.7 m. The measurement was performed at a sampling distance of 1 cm, starting from Tx–Rx separation of 3 m up to 3.31 m, which corresponds to 32 samples.

**Table 1:** Measurement Parameters.

Parameter	Value
**Range of frequency**	**3.1–5.3 GHz**
**Bandwidth**	**2.2 GHz**
**Transmit power**	**-14.5 dBm**
**Tx and Rx antenna gains**	**3 dBi**
**Samples per snapshot**	**1632**
**Sampling time**	**61 ps**
**Open Environment Tx antenna height (LOS, NLOS)**	**(2.0 m, 1.5 m)**
**Open Environment Rx antenna height (LOS, NLOS)**	**(1.7 m, 1.45 m)**
**Outdoor Corridor Tx antenna height (LOS)**	**(2.5 m)**
**Outdoor Corridor Rx antenna height (LOS)**	**(1.7 m)**

### The Evaluation Criterion

The proposed prediction method is evaluated based on the normalized mean square error (NMSE) between the predicted and measured WB-CIR taps given by:

$$E\left(p\right)=\frac{1}{K}\sum_{i=1}^{K}\frac{{\left(a_{w_{i}}\left(p\right)-{\tilde{a}}_{w_{i}}\left(p\right)\right)}^{2}}{{\left(a_{w_{i}}\left(p\right)\right)}^{2}}$$ (12)

Evaluating the method by predicting the WB-CIR at *L* − *p* location indexes, the average NMSE is computed using:

$$E_{avg}=\frac{\sum_{i=p}^{L}E\left(i\right)}{L-p}$$ (13)

Another approach that is used to evaluate the proposed model and prediction method is to use the estimated propagation channel parameters. The normalized path gain, maximum excess delay and root mean square (RMS) spread propagation parameters are estimated and compared for the predicted and measured CIRs in a particular location. The normalized path gain is estimated from CIRs, which is normalized based on the maximum gain of the path in MPCs (LOS path in LOS scenario and dominant path in NLOS scenario). The maximum excess delay is the delay of the path that has a 10% gain over the strongest path in one particular CIR. The RMS delay spread is calculated from the power delay profile (PDP), where the PDP of the particular CIR is given by:

$$p(t,\tau )={\left|h(t,\tau )\right|}^{2}$$ (14)

The RMS delay spread is defined by the second central moment of the (PDP) as [21]:

$$\tau_{rms}=\sqrt{\bar{\tau^{2}}-{\left(\bar{\tau_{m}}\right)}^{2}},$$ (15)

where $\bar{\tau^{2}}$ is the second moment of the PDP and is given as:

$$\bar{\tau^{2}}=\frac{\sum_{k}p\left(\tau_{k}\right)\cdot {\left(\tau_{k}\right)}^{2}}{\sum_{k}p\left(\tau_{k}\right)},$$ (16)

and $\bar{\tau_{m}}$ is the mean excess delay, also given as:

$$\bar{\tau_{m}}=\frac{\sum_{k}p\left(\tau_{k}\right)\cdot \tau_{k}}{\sum_{k}p\left(\tau_{k}\right)},$$ (17)

where *p* and *τ* are the power and delay of the *k*-th path, respectively.

### The Complexity Evaluation

Since there are *L* delay bins to be predicted for UWB CIR at spatial location *p* + 1, the complexity of RLS predictions becomes O(*M*^2^*L*). By reducing the number of delay bins to only *K<L* window taps, the complexity of the RLS prediction technique is reduced to O(*M*^2^*K*). As the proposed prediction technique predicts three filter coefficients of the current and neighboring windows, its complexity is three times higher than that of O(*M*^2^*K*).

### Results and Analysis

Based on the conducted measurement at Tx–Rx spatial distances of 3.00–3.31 m, we have *p* = 32 sets of measured CIRs. Each CIR consists of 1632 bins. One bin has duration of 61 ps, which is the optimized locked sampling time for the PulsON^®^410 receiver to capture the measurement pulse signals [20]. (Fig C in S1 Text) (S1 Text). Collect Data Procedures and Post Processing). This sampling time is higher than the Nyquist rate; hence, the bin resolution of the measurement is small to ensure that all MPCs are resolvable. Using the captured 1632 bins of CIRs, the size of the first window is set to 1 bin, and the size of the remaining windows *w* = 2,….,*W* is set to 7 bins. As a result, there are a total of *W* = 234 non-overlapping windows, which corresponds to a reduced WB-CIR with 234 taps. Here, the windows have equal size of *1/B*, where *B = 2*.*2* GHz is the bandwidth used in the measurement. The *1/B* size of the window is the minimum resolution required to maintain the channel characteristics; i.e., the second-order statistics such as root mean square (RMS) delay spread and maximum excess delay remain the same [18].

**Fig 3:**
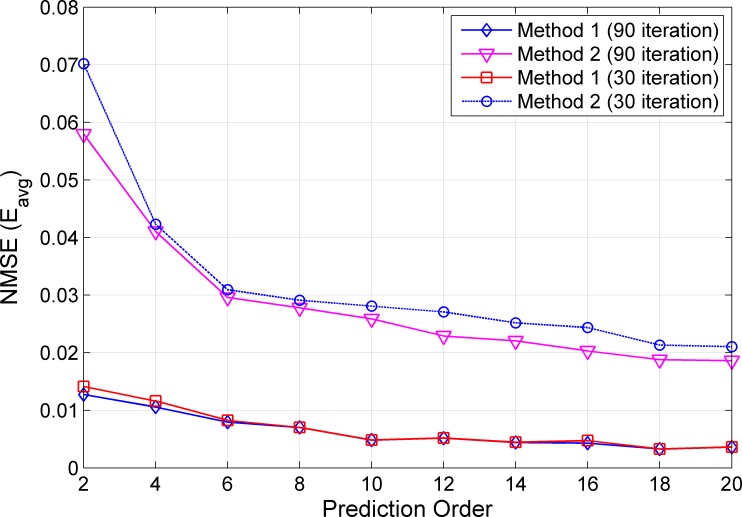
Average NMSE for LOS Scenarios in the Open Environment.

The generated WB-CIRs are then used for predictions using RLS algorithms of order 5, 10 and 15. For computing the average NMSE *E*_*avg*_ in (13), the proposed method is used to predict the WB-CIRs at several Tx–Rx location indices. For example, using a prediction order of 5, the WB-CIR prediction is carried out for 27 spatial locations of Tx–Rx distances from 3.05 m to 3.31 m. Note that we can predict the WB-CIR only at the spatial locations starting from 3.05 m because the RLS algorithm of order *M* = 5 requires five previous WB-CIRs of locations from 3.00 to 3.04 m. For comparison, we predict WB-CIRs using the RLS algorithms that consider only the previous *M* taps of WB-CIR from the current window (conventional method). Here, this benchmark method does not consider the tap correlation across the windows because the modeled AR process uses previous WB-CIRs only from the current window. We refer to the proposed and benchmark methods as Methods 1 and 2, respectively. Fig 3 shows the average NMSE *E*_*avg*_ of Methods 1 and 2 using RLS algorithms with *S* = {30, 90} iterations and prediction orders *M* = {2,4,…,20} for LOS scenarios. In general, the prediction errors improve when larger prediction orders are applied for both methods. Using the RLS algorithm with the same prediction order and iteration, Method 1 achieves smaller prediction errors compared with Method 2. Note that Method 1 requires the computation of three RLS filter coefficients; hence, computing each filter coefficient using RLS algorithms with lower *S = 30* iterations produces a total of 90 iterations for Method 1. Under similar computational complexity measured by the total number of RLS iterations, Method 1 with *S = 30* iterations still outperforms Method 2 with *S = 90* iterations. For prediction order *M = 10*, Method 1 with approximately *E*_*avg*_ = 5×10^−3^ is improved by approximately 80% in terms of prediction error compared with Method 2 with approximately *E*_*avg*_ = 2.6×10^−2^. Another advantage of Method 1 is that it is approximately three times faster than Method 2 because the three filter coefficients can be computed simultaneously with fewer *S = 30* iterations.

**Fig 4:**
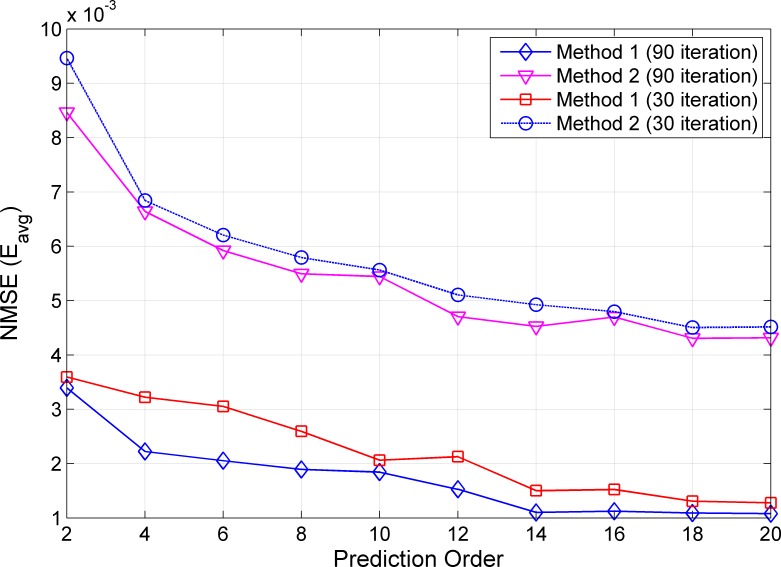
Average NMSE for NLOS Scenarios in the Open Environment.

Next, we present the average NMSE *E*_*avg*_ of Methods 1 and 2 using RLS algorithms with *S = {30*, *90}* iterations and prediction orders *M* = {2,4,…,20} for NLOS scenarios in Fig 4. We see that *E*_*avg*_ improves when larger prediction orders or higher RLS iterations are used for both methods. Similar performance, as in LOS conditions, can be observed for NLOS scenarios where Method 1 outperforms Method 2 for the three considered prediction orders. Comparing the two methods at similar computational complexity, Method 1 with *S = 30* achieves an average NMSE of approximately 2.0×10^−3^, whereas Method 2 with *S = 90* performs at approximately 5.4×10^−3^, for the prediction order of *M = 10*. This gives an improvement of approximately 63% when adopting our proposed Method 1.

**Fig 5:**
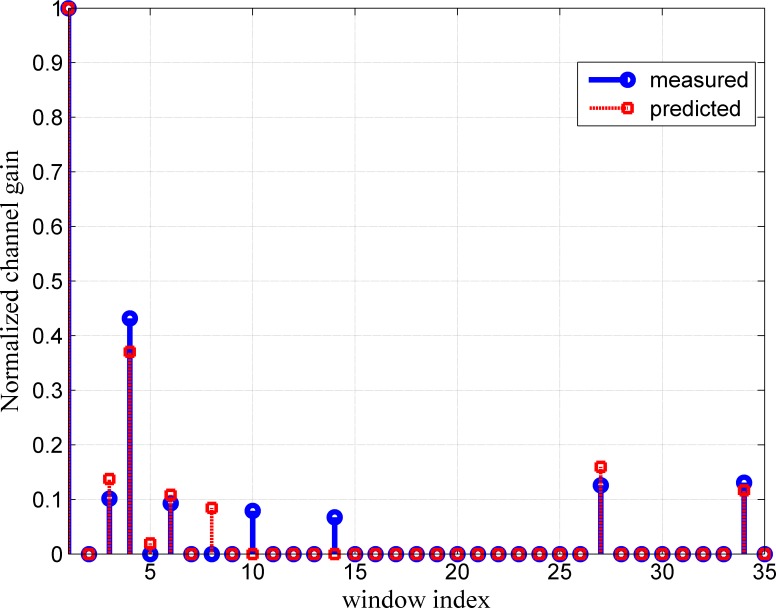
Channel Path Comparison for Measured and Predicted Channels in LOS Scenario at 3.11 m Tx–Rx Separation Distance.

**Fig 6:**
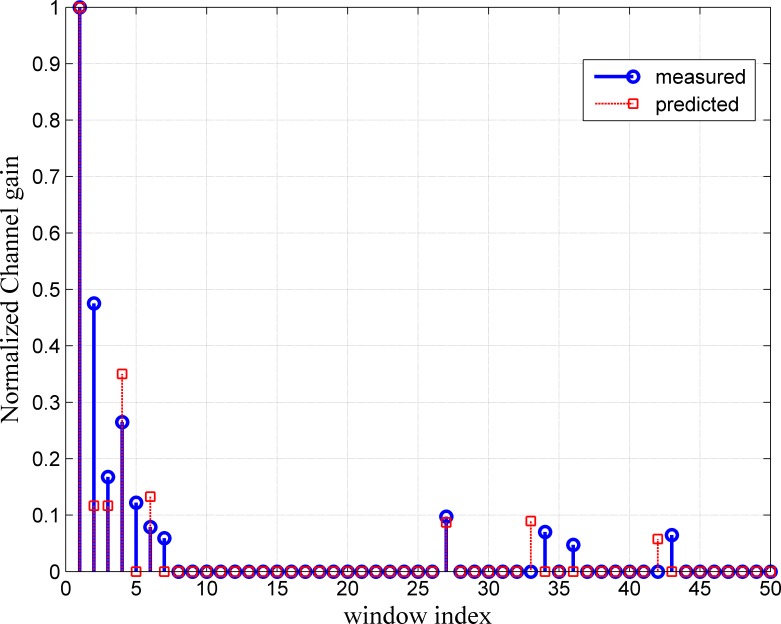
Channel Path Comparison for Measured and Predicted Channels in NLOS Scenario at 3.11 m Tx–Rx Separation Distance.

The results of the prediction method are also evaluated by comparing the predicted and measured channels in terms of the maximum excess delay, RMS delay spread and path gain of the channel. Fig 5 shows that the predicted channel almost totally matches the measured channel for the first prediction horizon (one step ahead prediction) in terms of the normalized path gain, number of paths and maximum excess delay. The RMS delay spread at different transmitter receiver separation distances is also compared. The RMS delay spread for the predicted CIR is almost the same as that of the measured channel at a separation distance of 3.1 m. The RMS delay spread values for a 3.1 m (10 cm from the first snapshot of CIR) Tx–Rx separation distance are 2.2 ns and 2.1 ns for the predicted and measured channels, respectively. In the NLOS scenario, the predicted channel especially differs from the actual measurement channel in the maximum excess delay as depicted in Fig 6. This difference in maximum excess delay will not affect the performance of the system since its value lower than the measured value.

**Fig 7:**
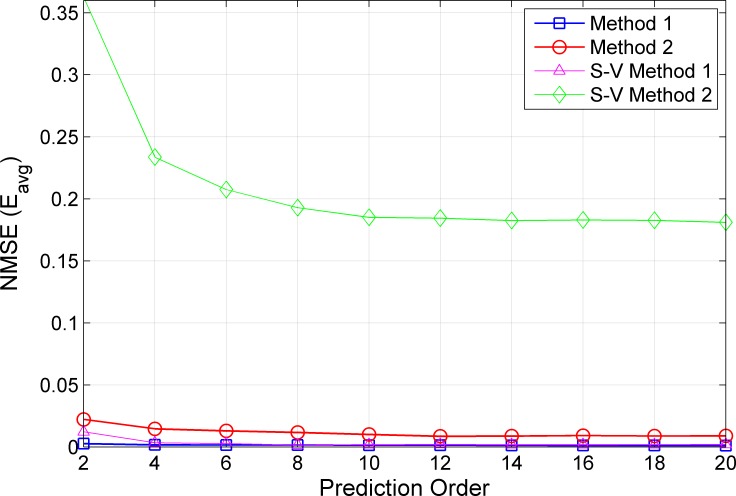
Average NMSE for LOS Scenarios in the Outdoor Corridor Environment.

To test the performance of the proposed prediction method, we used different datasets collected from a dense multipath environment in an outdoor corridor as described in the measurement and environment section (S1 Text). Fig 7 shows the average NMSE of the proposed prediction Methods 1 and 2 using RLS algorithms with 30 iterations and different prediction orders for LOS scenarios in an outdoor corridor environment. Method 1 with *S* = 30 achieves an average NMSE of approximately 1.0×10^−3^, whereas Method 2 with *S* = 90 accomplishes approximately 1.0×10^−2^ for a prediction order of *M* = 10. This gives an improvement of approximately 90% when adopting our proposed Method 1.

Fig 7 also illustrates the performance of our proposed Method 1 under a well-known Saleh-Valenzuela (S-V) channel model [17,22,23]. For the prediction order M = 10 and iteration S = 30, Method 1 achieves a lower average NMSE of approximately 0.002, whereas Method 2 performs at approximately 0.2. This gives an improvement of approximately 95% when adopting the proposed Method 1.

**Fig 8:**
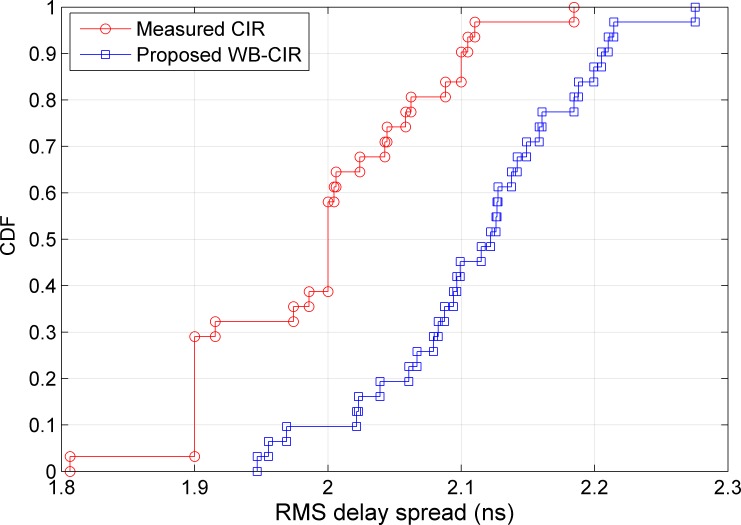
CDF of the RMS Delay Spread for the Open Environment.

**Fig 9:**
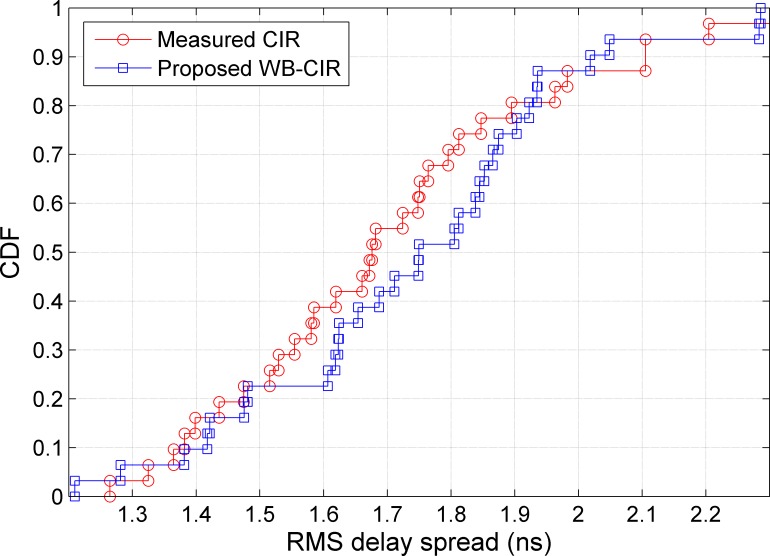
CDF of the RMS Delay Spread for the Outdoor Corridor Environment.

In addition, the RMS delay spread is investigated in this work using the proposed WB-CIR and comparing it with the measured one. It is used to estimate the maximum data rate for transmission [24]. The maximum transmission rate is inversely proportional to the RMS delay spread [25]. The cumulative distribution functions (CDF) of the RMS delay spreads for the measured and proposed CIR are provided for two different datasets as shown in Figs 8 and 9. For the studied open environment, Fig 8 shows that 90% of the RMS delay spread values for the measured CIR and proposed WB-CIR are less than 2.1 and 2.2, respectively. The average RMS delay spread values for the measured and proposed CIRs are 2.0 and 2.1, respectively. On average, using the measured CIR gives a data rate of approximately 500 Mbps, whereas the proposed method performs at approximately 476 Mbps. For the studied outdoor corridor environment, 80% of the RMS delay spread values are less than 1.9 ns for both the measured and proposed CIRs, with average RMS values of 1.7 ns as shown in Fig 9. This implies that the average RMS delay spread for the proposed method is totally matched with the measured CIR. The average data rate for both is 588 Mbps.

## Limitations and Future Works

The main objective of the proposed method is to model and predict the channel impulse response of the UWB channel. As a proof of concept, in this work, the size of each window is set only to the inverse of the transmitted bandwidth (*1/B*) because of the static environment measurement. In real applications—for example, when considering mobility scenarios, the optimal window size depends on the changes of the channel. The results also show that the proposed method works well within stationary scenarios.

As a future work, an improved model considering the mobility scenario and dynamic window size will be applied for an ultra-wideband channel in millimeter-wave frequency bands such as 28 GHz and 38 GHz that represent the candidate bands for the 5G wireless network [26,27].

### Conclusion

This work presented a prediction method for time-varying UWB CIRs that exploits the correlation between delay bins using windows. By considering the sparsity of UWB channels, we first proposed a simplified WB-CIR to represent the high temporal resolutions of UWB channels. The prediction method then tracked the evolution of WB-CIR taps in window *w*_*k*_ using three RLS coefficient filters computed from the observed channel taps of the left *w*_*k*−1_, the right *w*_*k*+1_, and the current *w*_*k*_ windows. The coefficient filter with the lowest RLS error is used for predicting the future WB-CIR. Compared with a conventional prediction method at similar computational complexity, our prediction method achieved better prediction errors with improvements of approximately 80% and 63% for LOS and NLOS scenarios, respectively.

## Supporting Information

S1 Table Raw Data for LOS. (ZIP)

S2 Table Raw Data for NLOS. (ZIP)

S1 Text Collect Data Procedures and Post Processing. (DOCX)
