# Supplementary material for: Window-Based Channel Impulse Response Prediction for Time-Varying Ultra-Wideband Channels
Source: PLoS One. 2016 Dec 19;11(12):e0164944. doi: 10.1371/journal.pone.0164944 (PMC5167263; doi:10.1371/journal.pone.0164944)
Supplement: S1 Text — (DOCX) [file pone.0164944.s003.docx]

**Supporting Information**

**A. M. Al-Samman^1,*^ , M. H. Azmi^1^, T. A. Rahman^1^, I. Khan^2^, M. N. Hindia^1^ and A. Fattouh^3^**

**^1^Department Wireless Communication Centre, Faculty of Electrical Engineering, Universiti Teknologi Malaysia, 81310 Johor**

**^2^Department of Electrical Engineering, Islamic University Madinah, Saudi Arabia**

**^3^Department of Computer Sciences, Faculty of Computing and Information Technology, King Abdulaziz University (KAU), Saudi Arabia, Jeddah,**

***Corresponding author**

**E-mail: Ahmedsecure99@gmail.com**

1. **Acronyms and Abbreviations**

The list of acronyms and abbreviations used in the main text and this Supporting Online Material:

| CIR: | Channel Impulse Response |
| --- | --- |
| CSI: | Channel State Information |
| LOS: | Line-Of-Sight |
| MPCs: | Multipath Components |
| NLOS: | Non-Line-Of-Sight |
| OFDM: | Orthogonal Frequency Division Multiplexing |
| PDP: | Power Delay Profile |
| RLS: | Recursive Least Square |
| RMS: | Root Mean Square |
| UWB: | Ultra-Wideband |
| WB-CIR: | Window-Based CIR |

1. **Measurement Setup and Environment**

Using time domain channel sounder, the data is collected in two different outdoor environments which has different structure. The first environment is chosen to represent the sparse environment where the multipath components are low (as open field environment, Fig 1), where some events like; weeding, sport and open party can be done on such environment. So, this study investigates changing on signals where users move freely. The second environment illustrates identical outdoor corridor beside the parking lots and low rise building environment (dense multipath environment, Fig 2). The choice of this environment is to depict an infostation scenario whereby mobile receiver download data as they may move along the corridor.

**2.1 measurement devices**

The UWB CIRs are collected from time domain measurements conducted in outdoor environments for LOS and NLOS scenarios by using a pair of PulsON^®^410 transceiver equipped with vertically polarized omnidirectional wideband (3.1-10.6 GHz) dipole antennas. The figures of the measurement devices and block diagram are shown in Figs. A and B. The details of the measurement devices can be found in [[1](#_ENREF_1)].


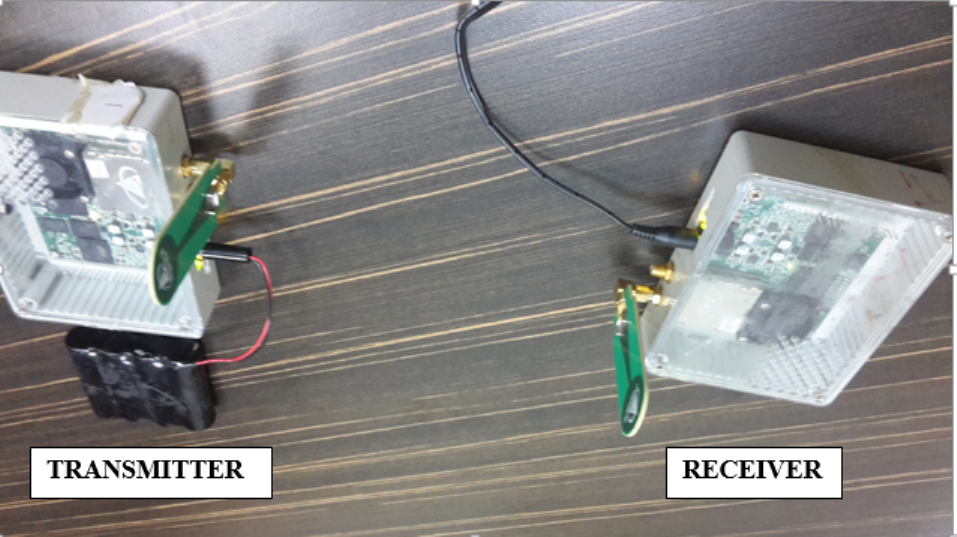


Fig A. The Measurement Equipment.


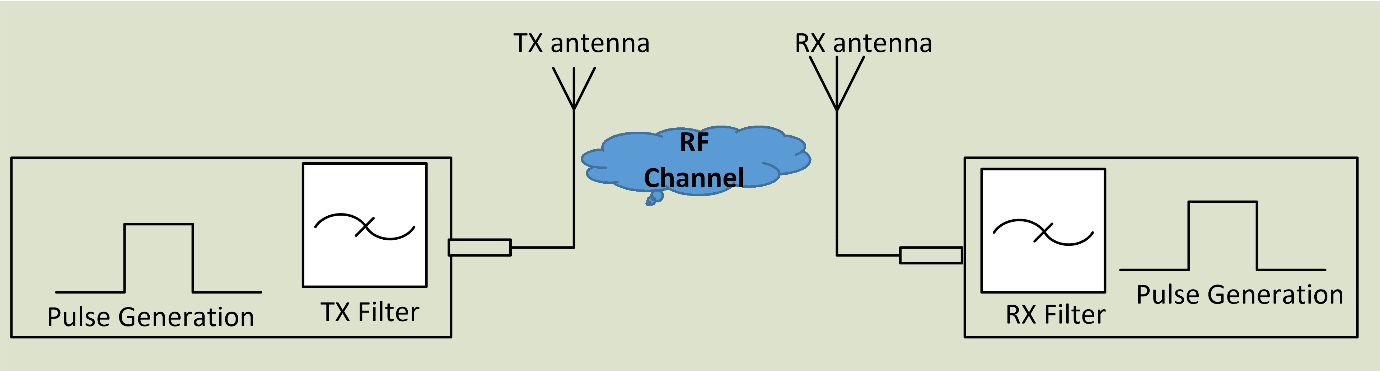


Fig B. The Block Diagram of the Measurement Setup.

**2.2 Measurement Environment**

The measurements were taken in two different cites inside Universiti Teknologi Malaysia. The first measurement camping is carried out in open place where the receiver was placed in front of the viewing platform, while the transmitter was moved away from the receiver as illustrated in Fig 1. At every transmitter movement of 1 cm, the CIRs were recorded starting from the Tx-Rx separation distance of 3.0 m till 3.31 m. The operator of the setup sits behind a brick column to ensure a stationary environment. In NLOS scenario, the directed path (LOS path) is blocked by a human obstacle with a height of 1.6 m stood in between the receiver and transmitter antennas at a 2 m distance from the receiver. The second measurements were conducted in outdoor corridor which represents the rich multipath environment as shown in Fig 2. This outdoor environment consists of a concrete floor and roofing, wooden columns used for the roofing, and many cars in the parking area. A grounded metallic sheet was placed between the person operating the attached computer system and the setup itself to ensure a stationary environment. The receiver antenna was located at a fixed point beside the metallic sheet while the transmitter part was, however, allowed to move. The transmission antenna height is 2.5 m, while the height of the receiver antenna is 1.7 m. The measurement was performed at a sampling distance of 1 cm, starting from Tx–Rx separation of 3 m until 3.31 m, which corresponds to 32 samples.

1. **Collect Data Procedures and Post Processing**

The data collection phase is done by step starting from the measurements phase until calling the data to Matlab tools box for extracting the channel impulse response as illustrated in Fig C. For future research, the raw data can be found in supporting files.

Fig C. The Block Diagram of the Measurement Phases and Post Processing.

The example of measurement procedure and post processing is shown in Figs D, E, F,G, H and L.


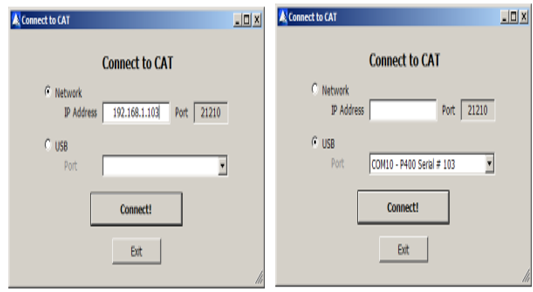


Fig D. The Connection Setup.


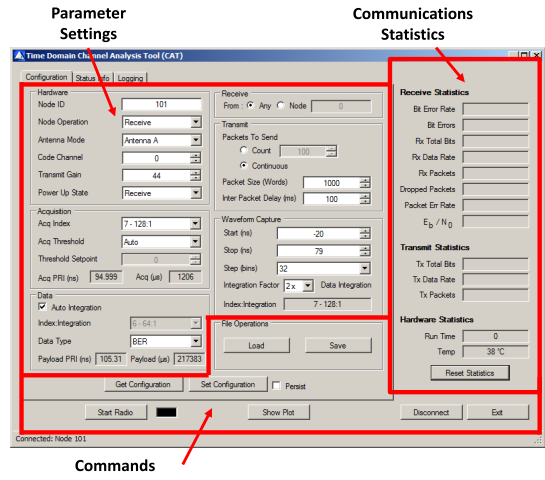


Fig E. The Parameter Setting.


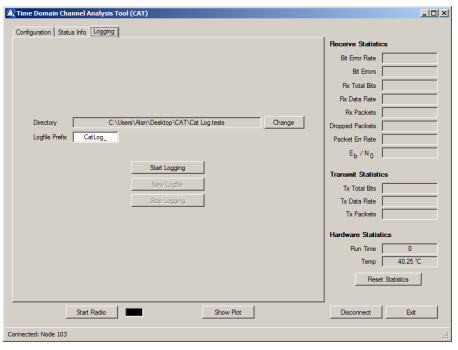


Fig F. The Data Collection.


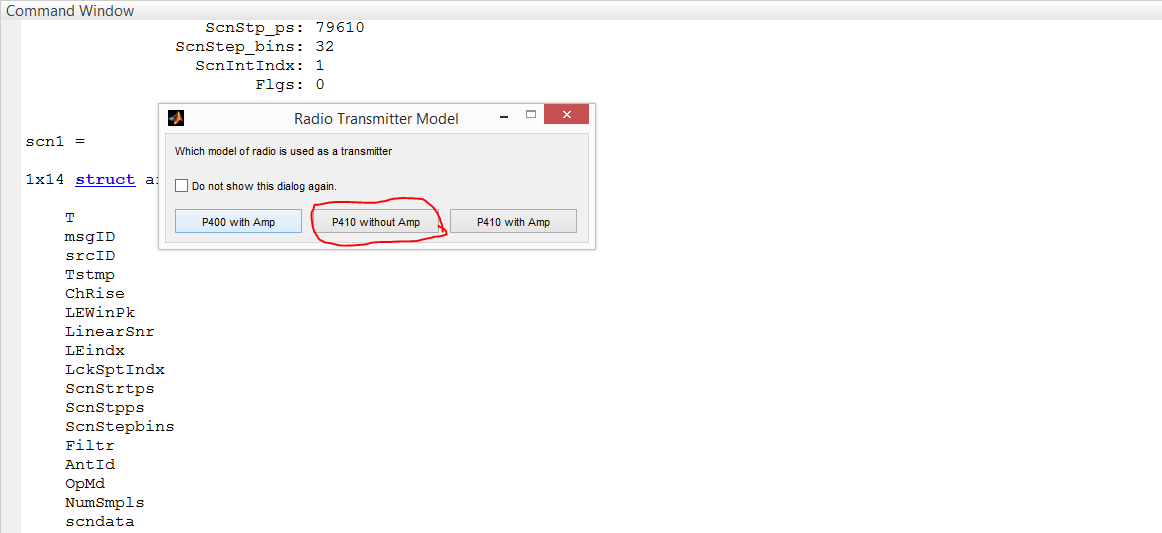


Fig G. The Post Processing.


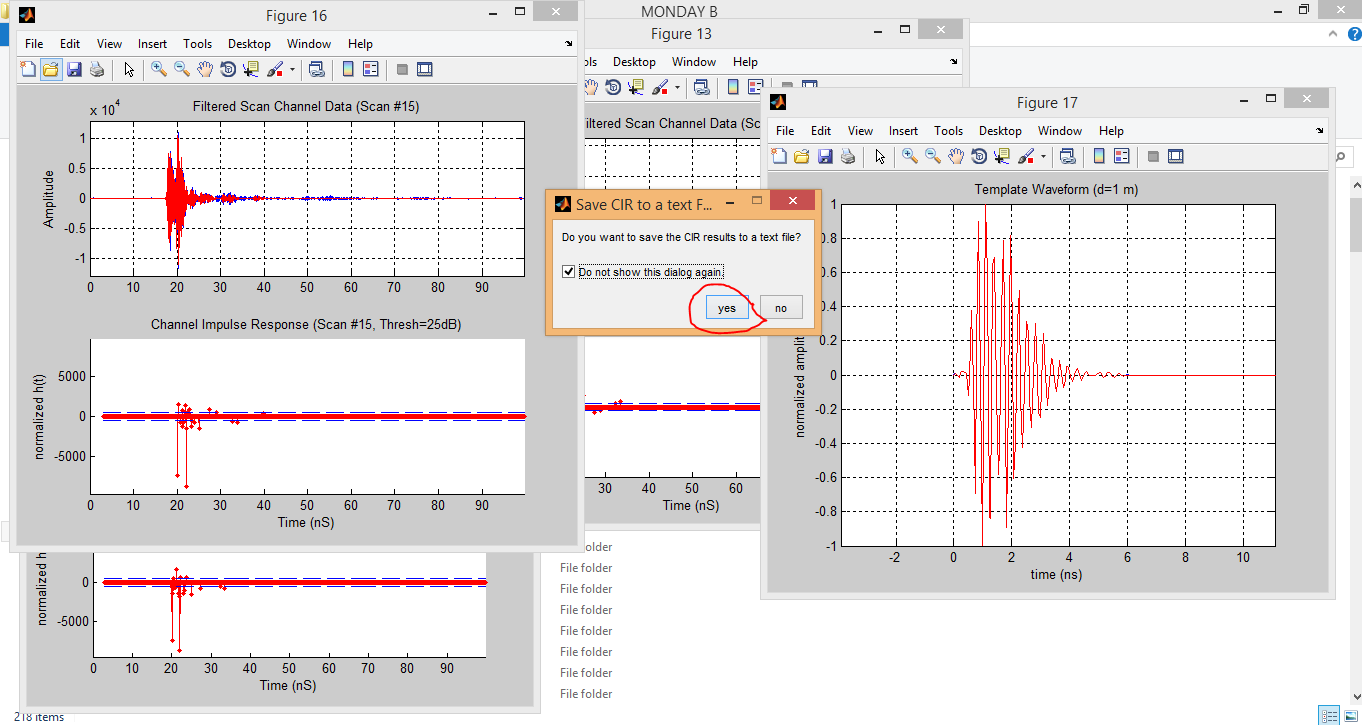


Fig H. The Channel Impulse Response Process.


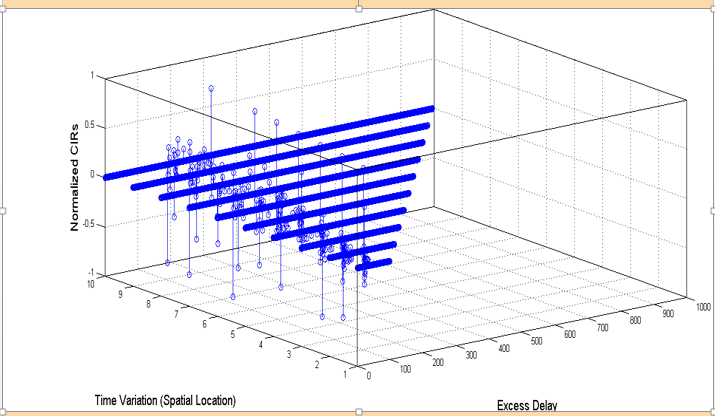


Fig L. The Time variant UWB Channel.

**References**

1. TIME DOMAIN, “CAT User Guide,” pp. 1–34, August 2012 [Online]. Available: http://www.timedomain.com.
